# Oedema in kwashiorkor is caused by hypoalbuminaemia

**DOI:** 10.1179/2046905514Y.0000000154

**Published:** 2015-05

**Authors:** Malcolm G. Coulthard

**Affiliations:** Great North Children’s Hospital, Newcastle-upon-Tyne, UK

**Keywords:** Oedematous malnutrition, Kwashiorkor, Oedema, Hypoalbuminaemia, Albumin, Nephrotic syndrome

## Abstract

It has been argued that the oedema of kwashiorkor is not caused by hypoalbuminaemia because the oedema disappears with dietary treatment before the plasma albumin concentration rises. Reanalysis of this evidence and a review of the literature demonstrates that this was a mistaken conclusion and that the oedema is linked to hypoalbuminaemia. This misconception has influenced the recommendations for treating children with severe acute malnutrition. There are close pathophysiological parallels between kwashiorkor and Finnish congenital nephrotic syndrome (CNS) pre-nephrectomy; both develop protein-energy malnutrition and hypoalbuminaemia, which predisposes them to intravascular hypovolaemia with consequent sodium and water retention, and makes them highly vulnerable to develop hypovolaemic shock with diarrhoea. In CNS this is successfully treated with intravenous albumin boluses. By contrast, the WHO advise the cautious administration of hypotonic intravenous fluids in kwashiorkor with shock, which has about a 50% mortality. It is time to trial intravenous bolus albumin for the treatment of children with kwashiorkor and shock.

## Introduction

Malnutrition in young children may lead to severe wasting alone (marasmus), or may be associated with oedema (kwashiorkor). The high mortality of severe acute kwashiorkor has changed little^1^ since it was first described in 1933,^2^ and about half of children who present today with shock still die. The World Health Organisation (WHO) recommend treating marasmus and kwashiorkor with the same fluid regimen when it is associated with shock,^3^ as if they shared precisely the same pathophysiology.

**Figure 1:**
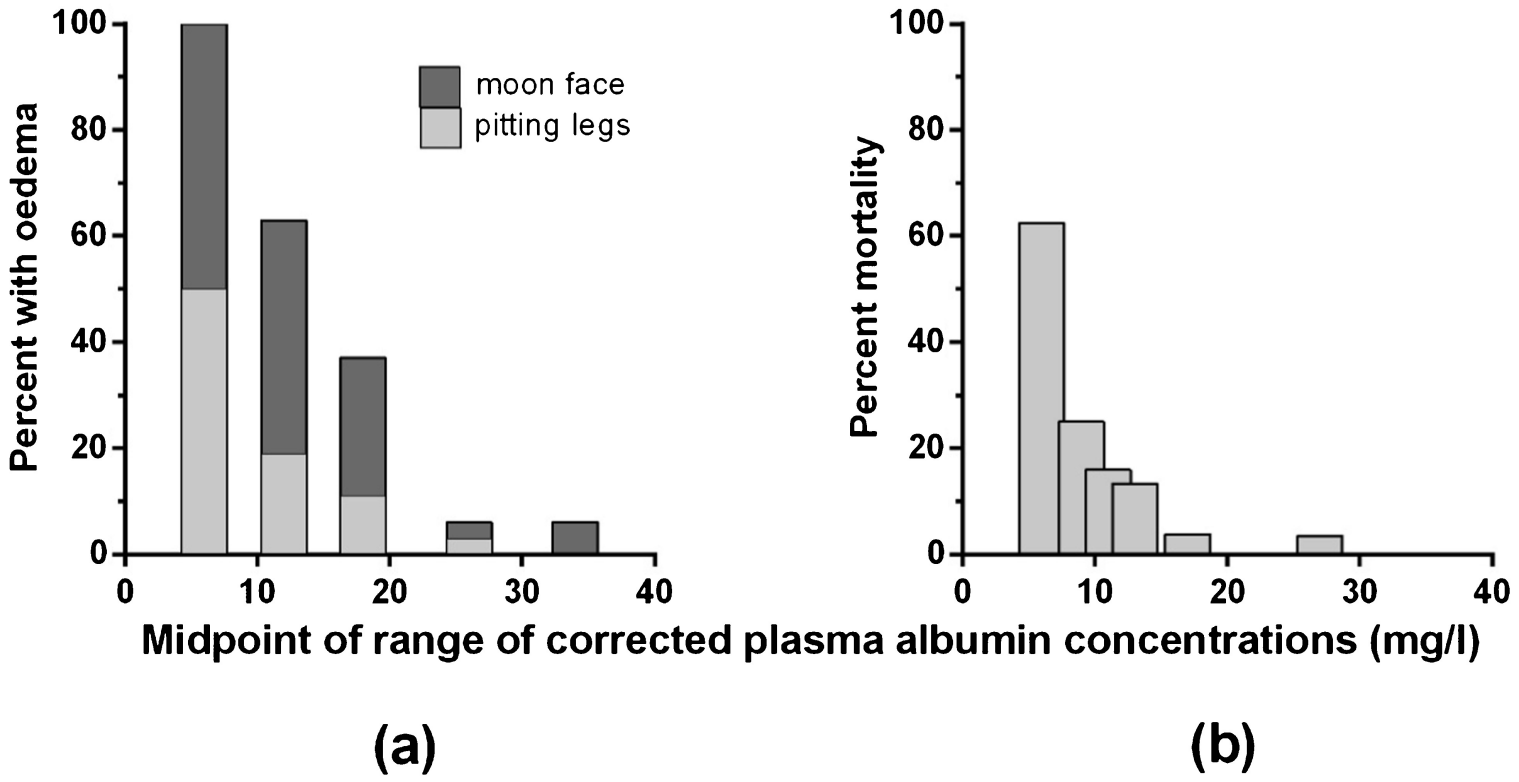
The relationships between the plasma albumin concentration in children with severe malnutrition and (a) the percentage chance of them having signs of oedema, and (b) their mortality risk, as identified by Whitehead^5^ and Hay^7^ in the early 1970s.

During the 1950s it was recognised that the presence of oedema in kwashiorkor was correlated with a very low plasma albumin concentration, presumably related to a dietary lack of protein.^4^ The closeness and importance of this link was identified in the early 1970s^5^^–^7 (Fig. 1), and its clinical predictive value has been confirmed since.^8^ However, in 1980 Golden and co-workers reported that there was not a causal link between the oedema of malnutrition and the low plasma oncotic pressure induced by hypoalbuminaemia,^9^ and this triggered extensive efforts to explain their disordered fluid physiology in other ways,^10^ including by the effects of specific micronutrient deficiencies, oxidant stresses and glutathione deficiency.^11^^–^13 He warned that the assumption that the oedema was directly related to hypoalbuminaemia could lead to therapeutic error.^12^ Here I review the pathophysiological evidence for a causal link between the oedema of kwashiorkor and hypoalbuminaemia, and consider what the therapeutic implications of this might be.

## What was the Evidence that Hypoalbuminaemia does not Cause Kwashiorkor Oedema?

Albumin is a relatively small protein, so it contributes disproportionately to the plasma oncotic pressure, and in health is its major contributor. Starling's equation^14^ explains how the movement and distribution of water between the plasma and tissue spaces of all tissues is physically regulated by the balance of hydrostatic and oncotic pressures across capillary blood vessel walls. However, Golden ruled out this mechanism as the primary cause for oedema in kwashiorkor by demonstrating that children who he treated with a relatively low protein diet showed marked clinical improvement and lost their oedema *before* their plasma albumin concentrations had risen.^9^

**Figure 2:**
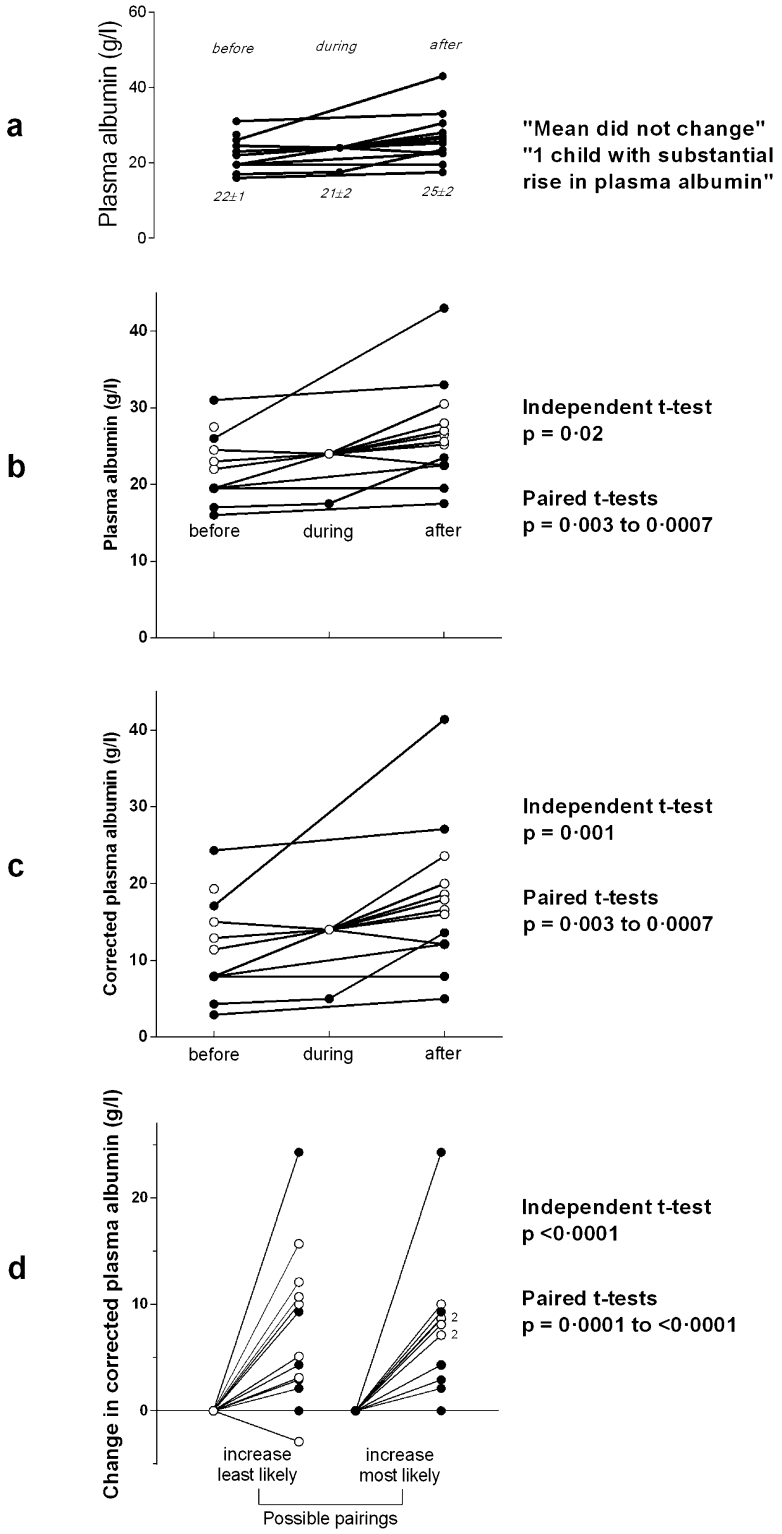
Graphs of the changes in plasma albumin concentrations in children on dietary treatment for kwashiorkor before, during and after the disappearance of oedema, from Golden et al, 1980.^9^ In graphs b to d, the filled circles represent paired pre- and post-treatment levels, and the open circles are cases where the correct patient pairing is not known.

The evidence for Golden’s unexpected finding was presented entirely graphically (Fig. 2a), without any corroborating statistical tests, accompanied by the observations that the mean albumin concentrations ‘did not change’, and that ‘only one child had a substantial rise’.^9^ The fact that these data were plotted in a physically small area (3×2 cm), with relatively wide aspect-ratio axes, and with descriptive text that happened to be slightly misaligned, may have contributed to the visual impression that the lines were approximately horizontal. By scanning and enlarging the figure and constructing a grid from the y-axis to obtain the numerical data, and re-plotting these values with a conventional aspect ratio and horizontal text (Fig. 2b), it can be seen that the plasma albumin levels *had* risen by the time that the oedema had improved. Furthermore, a two-tailed independent t-test confirms that this was a statistically significant rise in the mean albumin level (*P* = 0.02). Paired values can be discerned for six of the 13 cases (denoted by filled circles), and by combining the remaining seven cases in every possible way, paired *t*-tests show that the true *P*-value was somewhere between 0.003 and 0.0007.

## Measuring Plasma Albumin Concentrations

Albumin concentrations can be measured accurately by using specific immunological assays that only respond to that particular protein, even at very low levels.^15^ However, these techniques are not suitable for routine laboratory analysis, and instead dye-binding is used to provide approximate measurements. These methods rely on the fact that proteins have negatively charged surfaces that bind readily to certain positively charged dyes such as bromcresol-green (BCG), and that gram-for-gram, albumin binds more avidly than most of the globulins. However, globulins do bind with BMG, so when the albumin levels are very low this causes the measurements to be disproportionately high. For example, a plasma with no albumin could be reported as having as much as 15 g/L.^16^ Claims that this imprecision and skewing can be minimised by technical changes to the methodology or other dyes have not been confirmed.^17^ I have therefore estimated the likely true albumin concentrations from Golden’s publication as 1.4× (BCG – 14).^17^ Albumin estimates made from total protein measurements and electrophoresis analysis fall approximately half way between the BCG and true values.

The impact of using corrected albumins instead of BCG or electrophoresis values can be seen in Figure 2c, which demonstrates just how severely hypoalbuminaemic these children actually were on arrival. Finally, the true impact on their plasma albumin levels of feeding these children is most obvious when its increase is plotted for the period when they lost their oedema (Fig. 2d). Here, the *P*-value for all of the possible *t*-test permutations reaches <0.0001.

## What do other Studies of Albumin Levels in Kwashiorkor Show?

**Figure 3:**
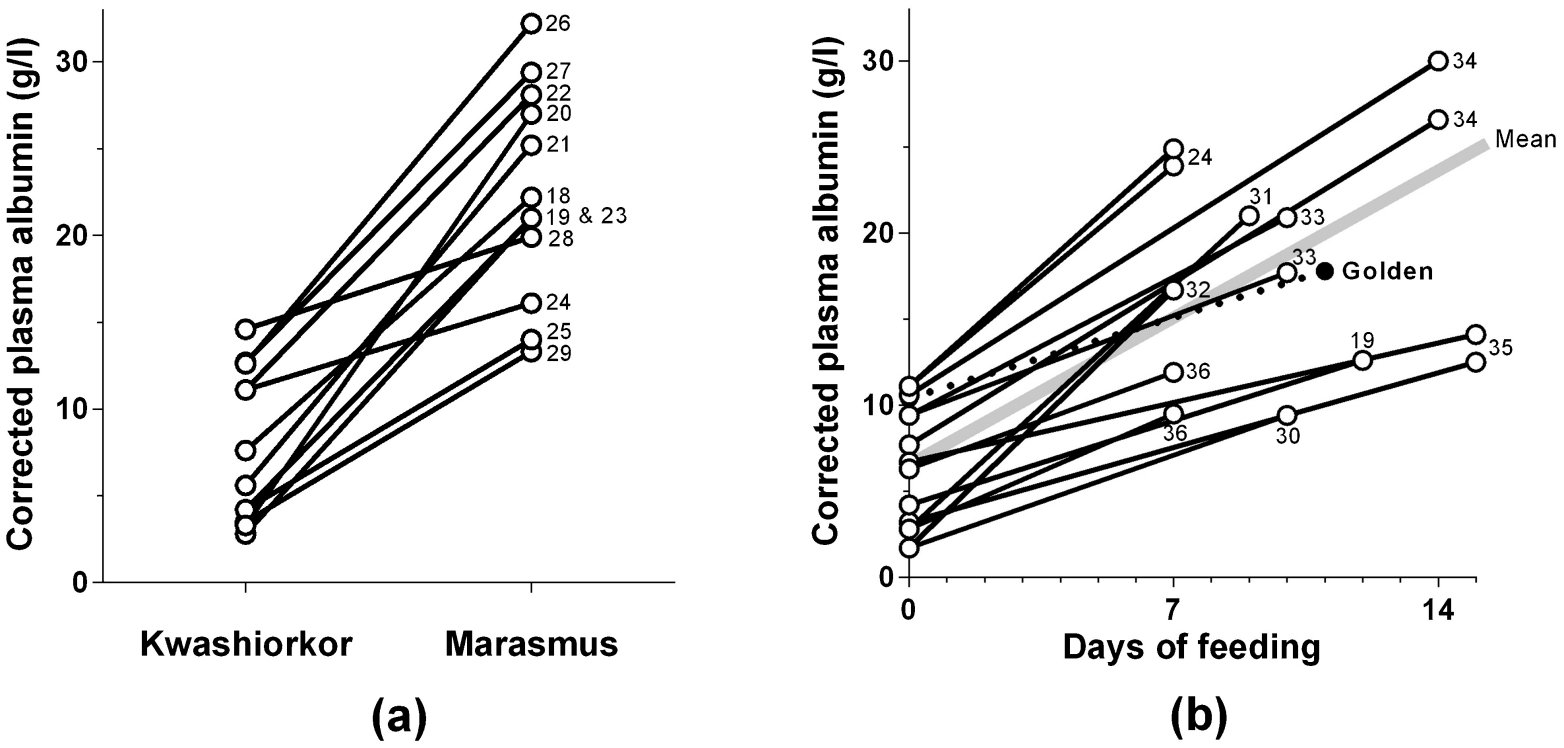
The corrected albumin concentrations measured in children with kwashiorkor (a) compared to children with marasmus in 12 studies, and (b) before and after feeding in 10 studies, four of which tested two different milks. Golden’s study detailed in Figure 2 is shown by filled circles and a broken line in graph (b), and the other lines are identified by the text references.

Though few other groups have presented their data in the same way as Golden, many other studies also recorded children’s plasma albumin concentrations when they presented with kwashiorkor and marasmus,^18^^–^29 or when those with kwashiorkor were given appropriate dietary treatment,^19^,^22^,^24^,^30^^–^36 in some cases whilst also comparing the efficacy of different milk formulas.^33^^–^36 Many of these studies were designed to elucidate the roles of other specific elements, such as vitamin deficiencies, but also included the albumin data, either as a list, a statistical parameter, or a plot, which allowed me to present them in a common graphic format in Figure 3. Both plots demonstrate just how low the true plasma albumin concentrations are in kwashiorkor. Figure 3a shows that in each study which included children with both marasmus and kwashiorkor, the mean albumin concentrations were consistently lower in kwashiorkor. Though there is some overlap between different studies, this may in part be owing to technical differences, such as measurement variations. In each study in which sufficient information was provided to make it possible to test the statistical significance of these differences, the *P*-values were all <0.05, and a paired *t*-test of the combined means gave a *P*-value of <0.0001.

Figure 3b shows that the plasma albumin rises promptly when appropriate milk feeds are introduced, with a mean daily increase of about 1.1 g/L. This compares to a mean daily increase of 0.7 g/L in Golden’s study.^9^ As with graph (a), every study in which the data could be evaluated statistically showed a significant increase on feeding, and the combined means showed a highly significant improvement. Some, but not all, of the reports indicated how long it took for the oedema to disappear, and these intervals were typically in the range of 6–12 days. These data provide no support for the hypothesis that the oedema resolved before the albumin rose.

## The Physiology of Kwashiorkor Looks a lot like Finnish Congenital Nephrotic Syndrome

The evidence I have reviewed thus far points to the pathophysiology of kwashiorkor being a combination of severe malnutrition and a low plasma oncotic pressure due to extreme hypoalbuminaemia. This closely resembles the pathophysiology of untreated Finnish congenital nephrotic syndrome (CNS), though of course the mechanism leading to them acquiring protein-energy malnutrition is very different. Infants with CNS simply cannot retain albumin, nor the smaller globulins, and waste vast quantities of energy. Today, children with CNS are managed very actively in developed countries, with drug treatment or unilateral nephrectomy to limit their proteinuria,^37^ or bilateral nephrectomy to stop it,^38^ followed by dialysis and transplantation. However, before this CNS was universally fatal by 18 months of age; children failed to thrive, and died of protein-energy malnutrition before they were old enough to develop renal failure.^39^ They were highly vulnerable to infections (despite penicillin prophylaxis), and had persistent oedema. Like children with kwashiorkor,^40^ they had markedly increased platelet stickiness. Low-dose aspirin is used to counter this in CNS, but of course if the same mechanism was responsible in kwashiorkor it would correct as the albumin rises with nutritional treatment. The two conditions also share similarly altered hormonal profiles.

Much attention has been drawn towards the low glutathione levels seen in kwashiorkor but not in marasmus.^12^ These have been interpreted as reflecting high levels of oxidant stress, and there has been speculation that this may be important in driving the development of the oedema. It was argued that the oedema of kwashiorkor could not be a consequence of hypoalbuminaemia as glutathione levels were said to be normal in nephrotic patients. However, this assertion was only based on one case in a study of children with kwashiorkor who had had a normal glutathione level and heavy proteinuria, who it was speculated “was probably a misdiagnosed nephrotic.”^11^ However, many studies have established that glutathione levels are low in persistent nephrotic syndrome.^41^,^42^ Although the exact relationship between reduced albumin and glutathione levels remains uncertain, they appear to be the consequence and not the cause of severe persistent low plasma albumin levels.

The major feature common to both kwashiorkor and CNS, however, is their disordered fluid balance physiology. Children with persistent nephrotic syndrome lose plasma water into the interstitium because of their low oncotic pressure, and as a consequence have chronic intra-vascular hypovolaemia. This induces avid water retention by an increased secretion of arginine vasopressin (antidiuretic hormone) in a non-osmolar response to hypovolaemia, and avid sodium retention by increased plasma renin activity and consequent secondary hyperaldosteronism, as well as by suppression of the release of the natriuretic peptides. This therefore leads to fluid retention and oedema, which is exacerbated if the child receives greater quantities of salt. The presence of oedema increases the interstitial pressure which therefore slows the accumulation of more oedema by balancing the Starling forces.^14^ Hence a stable situation evolves in which the child is persistently intra-vascularly hypovolaemic, has constant oedema, typically has a normal blood pressure, and has a tendency to slight hyponatraemia. Reducing the salt intake usually moderates the oedema, and there is a constant vulnerability to be ‘pushed over’ into frank clinical hypovolaemia with mild additional stresses to fluid balance, such as a bout of diarrhoea.

**Figure 4:**
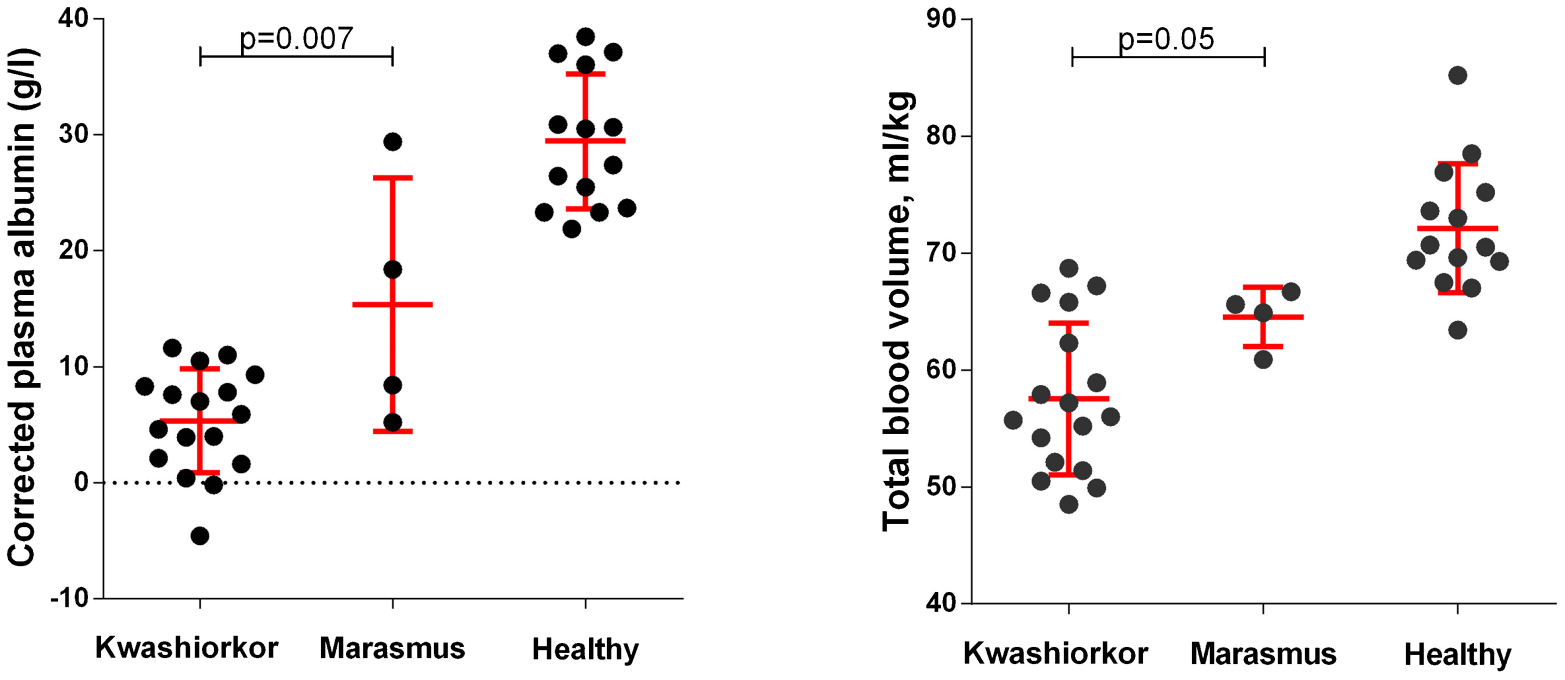
Corrected albumin concentrations and total blood volume measurements in children with kwashiorkor, marasmus, and healthy controls, from Viart.^43^ The malnourished children selected for this comparison were aged <3 years, with kwashiorkor defined as having ≧2+ oedema, and marasmus as having 0 or ± oedema. The error bars show the mean and standard deviation values.

Children with kwashiorkor are also markedly hypovolaemic and respond hormonally to this in the same way as nephrotic children. Viart demonstrated that children with severe malnutrition had a reduced blood volume compared to controls by re-injecting them with their own ^51^Cr-labelled red blood cells.^43^ He did not separately analyse children with marasmus and kwashiorkor, but his published data has allowed me to compare the albumin concentrations and total blood volumes (ml/kg of oedema-free weight) of children aged <3 years with either ‘0 or ±’ oedema (marasmus, *n* = 4) or with at least ‘++’ oedema on presentation (kwashiorkor, *n* = 17). The mean blood volume in marasmus is 90% of normal values, and in kwashiorkor it is just 80% (Fig. 4). Children with kwashiorkor also respond with very high vasopressin levels, which are higher than seen in marasmus, and which fall back to normal after loss of oedema following therapeutic feeding.^44^ Similarly, plasma renin activity is much higher in kwashiorkor than in control children, and highest by far in those who died acutely.^24^,^45^ Although these data fit precisely the physiological pattern of persistent nephrotic states, the hormonal changes confounded their authors at the time because they believed then that children with kwashiorkor were hypervolaemic. This was because early workers who attempted to measure the blood volume in children^46^ and animals^47^,^48^ with malnutrition measured the albumin space rather than the red cell space,^43^ despite them having clinical or sub-clinical oedema.

Although the body responds very quickly to the well described hormonal signals that are triggered by hypovolaemia, these immediate physiological adaptations are not the only ones that may occur, and prolonged exposure induces morphological and functional changes to the kidney.^49^ This means that patients with impending hypovolaemia from persistently low albumin concentrations are better able to produce concentrated urine and will be less likely to be so markedly oedematous as those who reach this state rapidly. This is why children with steroid-sensitive nephrotic syndrome (‘minimal change disease’) who present or relapse suddenly after an immunological stimulus may develop quite severe oedema initially, which may then lessen or even disappear prior to their loss of proteinuria as their renal functional capacity increases. However, this further up-regulation does not occur in kwashiorkor or CNS, as these adaptations will have already taken place.

## What are the Implications of this for Fluid Treatments for Children with Severe Acute Malnutrition?

All children who present with severe acute malnutrition may have serious complicating factors, but those who are not shocked are overwhelmingly likely to survive if they are treated according to WHO guidelines.^3^ For this group, differentiating between marasmus and kwashiorkor, and having a precise understanding of the physiology of oedema development, has little clinical relevance. However, it makes a vital difference when it comes to treating malnourished children who also have shock. Marasmic children, whose hypovolaemic shock is caused by an acute loss of salt and water uncomplicated by hypoalbuminaemia, then require an intravenous infusion of sufficient isotonic fluid to promptly restore the circulating blood volume. This allows oxygen delivery and perfusion of the organs,^50^ without perturbing the intra:extra-cellular tonicity gradients and thereby disrupting the volume and functioning of the body’s cells. A rapid 20-ml/kg bolus of an isotonic fluid with glucose, repeated as necessary, would fulfil these logical and physiologically-based criteria.^50^

By contrast, children with severe albumin deficiency from any cause continuously ‘struggle’ physiologically to maintain their blood volume by driving hormonal pathways that are normally only called upon in a crisis. They have no mechanisms in reserve; the mildest extra stress can rapidly precipitate severe shock. If that child happens to be one with CNS in a developed country, they will receive a prompt intravenous albumin infusion, and almost at once their signs of shock will wane as interstitial fluid is drawn into their blood vessels. A dose of frusemide administered soon after this will prevent rebound hypervolaemia and pulmonary oedema. They will mobilise large quantities of oedema as urine, re-establish a stable circulation, and will have a virtually guaranteed survival. However, if that same child was treated with just 30 ml/kg over 2 hours of half-strength Darrow's solution with 5% dextrose (hypotonic crystalloid; sodium 61 mmol/l), they may show a transient improvement as the fluid was delivered, but they would then deteriorate as the water leaked away into the tissues, and would have a high chance of dying. Yet this is what is recommended for shocked children whose hypoalbuminaemia happens to be caused by kwashiorkor.^3^ No distinction is made by the WHO between managing shock in marasmus and kwashiorkor, despite the fact that mortality is linked directly to the degree of oedema^2^,^5^ and hypoalbuminaemia.^8^,^24^ For this group, the mortality remains at around 50% in many parts of the world.^51^

The adoption of relatively conservative resuscitation fluid volumes for malnourished children has been driven in part by the concerns that larger quantities may precipitate congestive cardiac failure. This followed the fact that some very anaemic children died of heart failure after a few days of apparently successful progress on a therapeutic diet which contained a high salt content.^52^ However, this did not prove to be a problem in a randomised controlled trial of standard *vs* greater volume resuscitation, despite the severe warnings that this is likely to happen.^51^,^53^ Indeed, Viart very clearly described children with kwashiorkor dying as if they were still hypovolaemic, with none showing any evidence of congestive failure.^43^

## Conclusion

The mistaken belief that the oedema of kwashiorkor is unrelated to profound hypoalbuminaemia, combined with an exaggerated concern about the risks of congestive cardiac failure, has resulted in guidelines for shock management that fail to address their physiological needs, and which has not reduced their high mortality rate. Rather, children with kwashiorkor and CNS share a similar pathophysiology; both are malnourished and verge on intravascular hypovolaemia due to hypoalbuminaemia, and can be readily precipitated into shock. Treating this with intravenous albumin is life-saving in CNS; treating it late with modest volumes of hypotonic fluid has a 50% mortality in kwashiorkor. It is time for a trial of acute intravenous albumin therapy in children with kwashiorkor-related shock.
